# Selective autophagy and Golgi quality control in *Drosophila*

**DOI:** 10.1080/15548627.2022.2098765

**Published:** 2022-07-12

**Authors:** Raksha Gohel, Ashrafur Rahman, Peter Lőrincz, Anikó Nagy, Gábor Csordás, Yan Zhang, Gábor Juhász, Ioannis P. Nezis

**Affiliations:** a School of Life Sciences, University of Warwick, Coventry, UK; b Department of Anatomy, Cell and Developmental Biology, Eötvös Loránd University, Budapest, Hungary; c Institute of Genetics, Biological Research Centre, Szeged, Hungary; d State Key Laboratory of Silkworm Genome Biology, Biological Science Research Center, Southwest University, Chongqing, China; e Lead contact

**Keywords:** autophagy, *Drosophila*, Golgi, Golgiphagy, LIR motif, LIR-motif docking site

## Abstract

The LIR motif-docking site (LDS) of Atg8/LC3 proteins is essential for the binding of LC3-interacting region (LIR)-containing proteins and their subsequent degradation by macroautophagy/autophagy. In our recent study, we created a mutated LDS site in Atg8a, the *Drosophila* homolog of Atg8/LC3 and found that LDS mutants accumulate known autophagy substrates and have reduced lifespan. We also conducted quantitative proteomics analyses and identified several proteins that are enriched in the LDS mutants, including Gmap (Golgi microtubule-associated protein). Gmap contains a LIR motif and accumulates in LDS mutants. We showed that Gmap and Atg8a interact in a LIR-LDS dependent manner and that the Golgi size and morphology are altered in Atg8a-LDS and Gmap-LIR motif mutants. Our findings highlight a role for Gmap in the regulation of Golgiphagy.

Autophagy is a highly conserved cellular recycling process in which cells target cargo such as old and damaged proteins and organelles for degradation via the lysosome. This process is essential for cellular maintenance. Autophagy is characterized by the sequestration of cargo into phagophores that mature into double-membranes vesicles called autophagosomes. Selective autophagy receptors (SARs) contain LC3-interacting region (LIR) motifs that interact with the LIR-docking site (LDS) on Atg8-family proteins (LC3 and GABARAP proteins in humans, and Atg8a/b in *Drosophila*). The Golgi apparatus is a multi-functional organelle necessary for protein trafficking, modifications and sorting; however, autophagic degradation of the Golgi (Golgiphagy) has not been well characterized.

Because Atg8a is the primary Atg8-family protein involved in autophagy in *Drosophila*, we investigated its potential interactors by generating a CRISPR mutant of Atg8a’s LDS (Atg8a^K48A,Y49A^) in *Drosophila* to interrupt its ability to interact with LIR-containing proteins (LIRCPs) [1]. The mutation was confirmed with genomic sequencing and the Atg8a^K48A,Y49A^ flies accumulate known autophagy substrates ref(2)P and key (kenny), as well as ubiquitinated proteins when analyzed through western blotting. Immunofluorescence showed that ref(2)P and ubiquitinated proteins accumulate and form aggregates in adult *Drosophila* brains. This suggested to us that mutation of the LDS in Atg8a interrupt the autophagic degradation of LIRCPs. The lifespan of Atg8a^K48A,Y49A^ flies is approximately halved compared to control flies, demonstrating the physiological importance of the LDS.

By conducting quantitative proteomic LC-MS/MS analysis, we identified proteins that accumulate in the heads of virgin male Atg8a^K48A,Y49A^ flies and identified 29 proteins that are significantly upregulated in expression in these flies compared to that of wild-type *w^1118^* flies. Of these proteins, ref(2)P is present as expected, and the *Drosophila* Gmap (Golgi microtubule-associated protein) is also present. *Drosophila* Gmap is a *cis*-Golgi protein that plays a role in anterograde transport and is bound to the Golgi via a GRIP-related Arf-binding (GRAB) domain. To verify the proteomics finding, we performed western blotting and found that Gmap does accumulate in Atg8a^K48A,Y49A^ flies, and immunofluorescence also shows an increase in Gmap and ubiquitin puncta in the brains of adult Atg8a^K48A,Y49A^ flies. It was also observed that the Gmap puncta are increased in size in Atg8a^K48A,Y49A^ flies, suggesting to us that selective autophagy regulates the size and morphology of the Golgi via Gmap.

To examine whether Gmap interacts with Atg8a via a LIR-LDS interaction, we used the iLIR software and found a predicted LIR motif, DEFIVV, at position 320–325 in the Gmap protein sequence. GST affinity isolations were then used which showed that the Atg8a^K48A,Y49A^ mutant protein exhibits decreased binding to Gmap. Point mutations in the Gmap LIR motif (F322A V325A) also demonstrate reduced binding to Atg8a. These results indicate that Gmap interacts with Atg8a via a LIR-LDS interaction.

To investigate if Gmap may be involved in selective autophagic degradation of the Golgi, immunofluorescence was performed on adult fat bodies of Atg8a^K48A,Y49A^ flies that had been starved, and we observed that Gmap colocalizes with Atg8a in control flies, an interaction that is reduced in Gmap LIR mutant flies generated by CRISPR. GM130 (Golgi matrix protein 130 kD) is also a cis-Golgi protein and was used as a Golgi marker that was found to accumulate in Atg8a^K48A,Y49A^ flies, suggesting that selective autophagy is involved in Golgi maintenance. RNAi was used to knock down Gmap production, which also demonstrates GM130 accumulation.

Further investigation of Gmap’s role in Golgiphagy was performed. We found that GM130 accumulates in Gmap LIR mutant flies, and immunofluorescence showed that the Golgi morphology is deformed and elongated in Gmap LIR mutant flies. Furthermore, transmission electron microscopy was used, and we found that the Golgi area and length are increased in Gmap LIR mutants, furthering our hypothesis that autophagy is involved in Golgi regulation through Gmap. Despite these changes in Golgi morphology, secretion defects are not found in both the Gmap LIR mutants and the Atg8a^K48A,Y49A^ mutants, indicating that Gmap’s LIR motif and its interaction with Atg8a’s LDS, are not essential for Golgi secretory function.

Based on these observations, we propose that Gmap mediates selective autophagic degradation of the Golgi. Many of the observations of the Atg8a^K48A,Y49A^ mutant flies are similar to that of the Atg8a protein null flies Atg8a^KG07569^, including accumulation of known autophagy substrates, presence of ubiquitin aggregates and accumulation of Gmap. The accumulation of these proteins is stronger in Atg8a^KG07569^ mutants than in Atg8a^K48A,Y49A^, which may be due to the presence of a ubiquitin interacting motif (UIM) docking site (UDS) in Atg8a. Although Gmap has not been reported to contain a UIM motif, other proteins may regulate its turnover via this alternative autophagic binding site. Furthermore, the interaction of Gmap and Atg8a is not completely abolished through the mutations of Atg8a’s LDS or Gmap’s LIR motif, suggesting that there may be additional motifs or binding sites. A slight increase in bulk (nonselective) autophagy is observed in the Atg8a^K48A,Y49A^ mutants, which may be related to the stabilization of the mutated Atg8a.

In summary, Atg8a’s LDS is required for the autophagic degradation of LIRCPs and the *cis*-Golgi protein Gmap is one of these LIRCPs that interacts with Atg8a, and when Gmap’s LIR motif is mutated, Golgi morphology is altered and Golgi markers accumulate. We propose that Gmap regulates the turnover of the Golgi apparatus via selective autophagy (Golgiphagy) through a LIR-LDS interaction. These findings highlight the physiological importance of selective autophagy in cellular maintenance and increase our understanding about Golgiphagy which has yet to be fully elucidated.
